# Multipolar interference for non-reciprocal nonlinear generation

**DOI:** 10.1038/srep25113

**Published:** 2016-04-29

**Authors:** Ekaterina Poutrina, Augustine Urbas

**Affiliations:** 1 Materials and Manufacturing Directorate, Air Force Research Laboratory, Wright Patterson Air Force Base, Ohio 45433, United States; 2 UES, Inc., 4401 Dayton-Xenia Road, Dayton, OH 45432, United States

## Abstract

We show that nonlinear multipolar interference allows achieving not only unidirectional, but also non-reciprocal nonlinear generation from a nanoelement, with the direction of the produced light decoupled from the direction of at least one of the excitation beams. Alternatively, it may allow inhibiting the specified nonlinear response in a nanoelement or in its periodic arrangement by reversing the direction of one of the pumps. These general phenomena exploit the fact that, contrary to the linear response case, nonlinear magneto-electric interference stems from a combination of additive and multiplicative processes and includes an interference between various terms within the electric and magnetic partial waves themselves. We demonstrate the introduced concept numerically using an example of a plasmonic dimer geometry with realistic material parameters.

Reciprocity is a unique property of light propagation^1^, serving as an underlying basis in any optical design. Even in a lossy system where time-reversal symmetry does not hold^2^, transmission of a light beam through a finite set of elements characterized by a symmetric and time-independent scattering matrix stays the same when reversing beam direction, or interchanging the source and the detector^3^,^4^,^5^. The fundamental nature of this phenomenon results, on the other hand, in unavoidable challenges when selectivity in the direction of light transmission is required.

One-way light propagation is indispensable for a variety of applications, including integrated optics and photonics, optical communications, and quantum computing, as it allows for the development of ultra-fast all-optic logic circuits. The current most common approach, relying on non-reciprocal Faraday rotation of the polarization plane^6^, requires the use of magneto-optic media with the application of a static magnetic field and is rather bulky, remaining incompatible with on-chip, moreover nanoscale applications, despite attempts to miniaturize it^7^,^8^,^9^,^10^. Alternative techniques avoiding the use of a magnetic bias field have to rely on either time-varying media or nonlinear effects. In contrast with linear scattering, the nonlinear response, in general, is not subject to the limitations imposed by the reciprocity theorem; in fact, a series of works^11^,^12^,^13^,^14^,^15^,^16^,^17^,^18^,^19^,^20^,^21^,^22^ have suggested designs that allow a non-reciprocal nonlinear optical transmission. Many of the proposed geometries rely on an asymmetric material composition along the propagation direction, exploiting the resulting longitudinal variation of the local field distribution within the structure^11^,^12^,^13^,^14^,^15^,^16^. However, such an approach is not entirely immune to a non-zero back-scattering noise transmission in the presence of a strong forward signal^23^. A set of works exploited the geometric asymmetry of nonlinear elements^17^,^18^,^19^,^20^,^21^,^22^. Although all of the proposed methods make the on-chip integration of non-reciprocal optical elements incomparably more feasible, the gap between the footprint of the geometries demonstrated in the optical domain and truly nanoscale sizes persists. In addition to incompatibility with nanoscale technologies, most of the proposed approaches are device-scale, larger than the working wavelength; thus, they lack the flexibility to serve as building blocks for engineering artificial media with an incorporated nonreciprocal response.

Light manipulation at the nanoscale, while providing a possible path for controlling interactions near an atomic-scale level, faces additional challenges unique to the microscopic nature of the processes involved. Not only the scattering response is subject to usual limitations due to the reciprocity theorem, but the directionality control is overall considerably reduced, due to the decrease in the degree of coherence occurring on interaction of even a highly coherent light beam with a nanoscale object^6^. Although focusing and re-routing light are routine macroscopic operations, diffraction dominates on a nanoscale. As a result, the interference between the multipolar modes produced on scattering by the nanoelement^24^,^25^,^26^ –a process inherent to nanoscale interactions–is at the heart of directionality control. The interference between partial waves of opposite parities (such as those of electric and magnetic dipolar modes) and of a comparable strength can strongly suppress linear scattering in one direction, either forward or backward, while nearly doubling it in the opposite direction^27^,^28^,^29^. Subject to the reciprocity theorem, however, the sides that are suppressed and doubled switch when the direction of the excitation beam is reversed. For example, a forward unidirectional scattering stays “forward” with respect to the direction of the excitation beam, rather than with respect to a fixed laboratory coordinate system. Hereafter, such a *reciprocal* directional scattering will be termed as “unidirectional”.

Similar to linear scattering, the nanoscale nature of the interaction prevents regular mechanisms, such as phase matching, from having an impact on the directionality of the nonlinear response. As a result, nonlinear generation from a nanoelement occurs nearly isotropically, for all nonlinear processes involved. Similar to the linear response, multipolar interference can take place between the partial waves in the nonlinearly generated field, thereby helping to restore directionality control. Indeed, the interference between the nonlinearly produced electric and magnetic partial waves was shown to induce some asymmetry between the “transmission” and “reflection” sides in the far field pattern of the second harmonic generated from plasmonic nanostructures, which has been subsequently used for the analysis of multipolar contributions to the nonlinear response^30^,^31^,^32^. Furthermore, the possibility of a complete suppression of the nonlinearly generated field in one of the hemispheres has been suggested theoretically^33^ and demonstrated experimentally^34^ to produce a unidirectional nonlinear generation in the microwave regime from deeply sub-wavelength metamaterial layers. The nonlinear multipolar response^35^,^36^,^37^,^38^,^39^ and phase-sensitive control of nonlinear scattering from nanoelements^40^ have also been actively discussed in relation to optical nanoantennas. The reported asymmetry in the far field pattern of the nonlinearly generated field was, however, entirely analogous to a similar phenomenon in linear scattering, in which the suppressed and doubled sides switch when the direction of the excitation is reversed.

As we show here, nonlinear multipolar interference offers capabilities fundamentally different from those provided by its linear counterpart. In particular, we show that it allows both a non-reciprocal and unidirectional nonlinear generation where the generation direction is preserved with respect to a fixed laboratory coordinate system when reversing the direction of the fundamental field. In contrast with the previous designs that ensured a non-reciprocal response via nonlinear interactions^11^,^12^,^13^,^14^,^15^,^16^,^17^,^18^,^19^,^20^,^21^,^22^, in the approach proposed here, non-reciprocity with respect to the direction of a subset of the fundamental excitation beams can be achieved with purely symmetric structures, both in terms of their geometry and material composition. Moreover, the proposed concept assumes an intrinsic non-reciprocal directionality of nonlinear generation from sub-wavelength structures. The latter opens a path for developing macroscopic media with a similar, inherently incorporated, non-reciprocal nonlinear response, where such structures are used as building blocks.

We also discuss the possibility that nonlinear multipolar interference can ensure a directionally selective inhibition of the nonlinear response for certain respective directions of the fundamental beams. We attribute the presented phenomena to the existence of common (electric or magnetic) pathways inducing Mie resonances via a nonlinear interaction. These pathways arise due to the multiplicative nature of the nonlinear response and result in the fact that switching the phase of *one* (electric or magnetic) of the fundamental field vectors can *simultaneously* change the phase of *all* (electric and magnetic) nonlinearly generated multipoles. Furthermore, we show that the interference can occur between various terms *within* the electric and magnetic (nonlinearly produced) dipolar modes themselves. As a result of this interference, a slight change in the *efficiency* of nonlinear generation occurs when the direction of *any subset* of the fundamental beams is reversed. Consequently, non-reciprocity in terms of ensuring just a change in the efficiency of nonlinear generation when reversing the direction of any subset of the fundamental beams is inherent to and expected in the nonlinear response of most nanoelements, even the symmetric ones, and for most nonlinear processes. Further, engineering a certain relation between various interfering terms within each (electric and magnetic) nonlinearly generated partial wave can allow the above-mentioned complete inhibition of a specified nonlinear response.

The proposed concept offers the flexibility of achieving all the presented phenomena via the response of sub-wavelength elements, thus allowing their use as building blocks in developing a nonlinear media with similar unique features. Both non-reciprocal directionality and inhibition of the nonlinear response require engineering the relative strengths of electric and magnetic partial waves, as well as the relative strengths of various terms within each of these modes. This balance of strengths and the resulting phenomena are not expected to occur in the nonlinear response of natural materials but, as we show here, can be realistically achieved by designing the multipole response of nanostructures. Combined with a strong enhancement of the nonlinear efficiency provided by resonant nanoelements^41^,^42^,^43^, the proposed concept can be used to engineer artificial nonlinear nanomaterials with an enhanced and inherently non-reciprocal response for a variety of applications in integrated optics and photonics.

## Results

**Figure 1:**
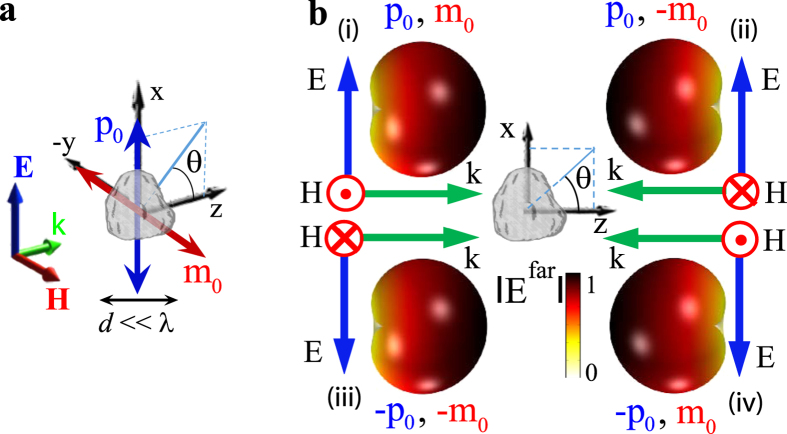
Linear magneto-electric interference. (**a**) Visualization of the coordinate system orientation used in equation (1). (**b**) Angular distribution of the norm of the scattered far field, produced by the interference between electric and magnetic dipolar modes induced in a nanoelement via a linear response, as shown in Fig. 1(a), for the four pump arrangements indicated in sets (i)-(iv). The matched strength of electric and magnetic modes is assumed in all cases. Reversing the direction of the incident field using arrangement (ii) or (iv) results in one of the vectors (electric or magnetic) of the excitation field having a phase that is opposite to that in set (i). This leads to a sign change before a single term in equation (1), resulting in the reversed directions of suppression and doubling of the scattered field, compared to set (i). In set (iii), the phases of oscillation are reversed simultaneously for both electric and magnetic dipolar modes, resulting in a preserved scattering direction.

In order to illustrate the general concept, consider a nanoelement that has a set of electric and magnetic dipolar modes induced along the *x* and *y* Cartesian axes, respectively, via an optical response to radiation incident along *z* direction, as shown in Fig. 1(a). Further assume that no other multipoles acquire significant strength within the same spectral range. The radiation from such orthogonal, spatially superimposed electric and magnetic dipoles results in the following angular distribution of the *E*_*θ*_ far field component of the electric field, scattered in the *xz* (*φ* = 0) plane^44^:


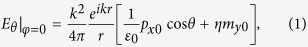


with angles *θ* and *φ* in a standard spherical coordinate. Here, *p*_*x*0_ (*m*_*y*0_) denotes the amplitude of the *x* (*y*) component of the induced electric (magnetic) dipole, 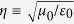 is the vacuum impedance, with *ε*_0_ and *μ*_0_ being the permittivity and the permeability of vacuum, *r* is the length of the radius-vector directed from the scatterer (located at the origin) to the observation point in the far field, and *k* is the wavenumber. One can show *E*_*φ*_ = 0 in the *φ* = 0 scattering plane, and the radial component *E*_*r*_ falls off in the far field.

Following equation (1), the radiation from the above set of dipoles can be interferometrically suppressed in either backward (*θ* = 180°) or forward (*θ* = 0°) direction when the strengths of the electric and magnetic dipolar modes are being matched with either the same (1/*ε*_0_ *p*_*x*_ = *ηm*_*y*_) or opposite (1/*ε*_0_ *p*_*x*_ = −*ηm*_*y*_) signs, respectively. These scenarios are known as the first and the second Kerker^24^ conditions. (Note that forward scattering suppression is subject to optical theorem limitations^25^,^26^,^44^).

While the Kerker conditions are often associated with matching the values of electric (*α*^*el*^) and magnetic (*α*^*m*^) polarizabilities of a nanoelement, as seen from equation (1), it is the strengths of the dipolar modes themselves (the latter related to the electric (**E**_**0**_) and magnetic (**H**_**0**_)) vectors of the incident field as 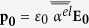 and 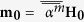 that regulate the resulting interference pattern (here and further bold font indicates vector quantities). Consequently, the direction of enhancement and suppression of the scattered field can be controlled by changing the sign of one of the polarizabilities, as well as by switching the phase of the inducing electric or magnetic field. In fact, this dependence on the phase of the excitation field vectors allows for a simple illustration of the reciprocity of linear scattering, even when a unidirectional response is achieved, as explained below.

### Linear multipolar interference: illustration of the reciprocity of linear scattering

Set (i) in Fig. 1(b) provides a numerical example of the backscattering suppression achieved when there is a match in strength between the dipolar modes oscillating in phase. Reversing the direction of the incident beam using, e.g., the arrangement shown in set (ii) or set (iv) from Fig. 1(b) leads to phase switching in one (either magnetic or electric) of the vectors of the excitation field, with respect to its phase in set (i). This, in turn, results in a sign change of a single term in equation (1), causing the doubling and suppression of the scattered field to switch directions, compared to set (i) in Fig. 1(b). As such, forward (backward) unidirectional scattering always stays “forward” (“backward”) with respect to the direction of incidence of the excitation beam, rather than to a fixed laboratory coordinate system. This makes the linear scattering unidirectional with respect to the incident beam, but not non-reciprocal.

In that vein, it is instructive to consider the arrangement in set (iii) in Fig. 1(b). In this case, the phase of oscillations of *both* electric and magnetic excitation field vectors is opposite to that in (i). This results in a *simultaneous* sign change before both electric and magnetic terms in equation (1) and, as follows from the equation, in a preserved direction of the scattered far field. The situation is somewhat similar to the “negative index” media scenario, in which the direction of energy propagation is preserved when the phase velocities of both the electric and magnetic partial waves are simultaneously reversed^45^,^46^. However, in the present case, such a reversal occurs due to the simultaneous phase switching of the electric and magnetic vectors of the incident field, rather than the simultaneously negative values of electric and magnetic polarizabilities.

### Nonlinear multipolar interference: proposed general concept

Radiation in one hemisphere can clearly be suppressed, whether the dipolar modes are induced through a linear or nonlinear interaction. In the latter case, it leads to an asymmetry in the generated far field pattern on the “reflection” and “transmission” sides–a phenomenon similar to the linear response and discussed previously in a series of works^30^,^31^,^32^,^34^. In contrast with the linear response case, however, as we show here, nonlinear multipole interference allows for a non-reciprocal nonlinear generation that preserves this asymmetry with respect to a fixed laboratory coordinate system when reversing the direction of the excitation beam(s).

#### Nonlinear dipolar modes as a result of interference between various effective hyperpolarizability terms

In the case of a nonlinear response, each of the dipolar moments can result from a sum of several terms^33^,^47^. The number of terms is dependent on the order of the nonlinear process. In the most general case, for the second order response at frequency *ω*_3_ = *ω*_1_ + *ω*_2_, assuming a monochromatic excitation, the relations can be written as follows:


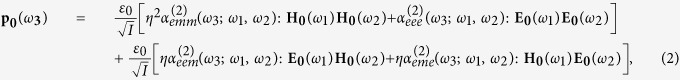



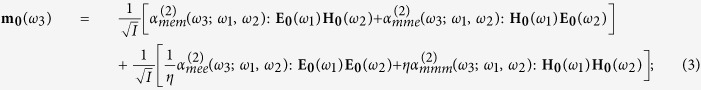


where *I* ≡ |**E**_**0**_(*ω*_1_)||**E**_**0**_(*ω*_2_)|, and **p**_0_ and **m**_0_ are vectors of the nonlinearly induced electric and magnetic dipole moments, respectively. The time-harmonic factor has been omitted, and the double-dot product denotes the summation over all Cartesian components. The subscripts “*e*” and “*m*” indicate the type (electric or magnetic) of dipolar modes that are participating in the interaction; the first subscript denotes the type of produced dipolar mode at *ω*_3_, and the second and third subscripts denote the types of contributing fundamental modes at *ω*_1_ and *ω*_2_, respectively. We emphasize that, noting a pure electric intrinsic response in the case of non-magnetic materials assumed here, the coefficients on the right hand sides of equations (2) and (3) represent the effective, rather than the intrinsic, hyperpolarizabilities. The origin of such effective nonlinear response is different for plasmonic or other nanoelements made of centro-symmetric materials, as opposed to those possessing an intrinsic second-order nonlinear response. Normalization has been chosen in order to keep the single units of [m^3^] for all hyperpolarizability types.

One can think of the various terms entering the right hand sides of equations (2) and (3) as of different pathways leading to the certain type (electric or magnetic) of the nonlinear response. Note that, in either equation (2) or (3), the terms in the first and second brackets comprise the sets of effective hyperpolarizabilities of two different types, polar and axial^33^,^48^, respectively.

As seen from equations (2) and (3), in addition to the interference between electric and magnetic modes, in the case of a nonlinear response, the interference can occur between various hyperpolarizability terms within each of these modes. One can also encounter a situation where electric and magnetic hyperpolarizabilities within the same symmetry group share the same (either electric or magnetic) pathway through the fundamental field of a given frequency. The existence of such shared pathways can allow for a non-reciprocity in the direction of nonlinear generation, as discussed below.

#### Non-reciprocity in the direction of nonlinear generation: proposed concept

Consider now a situation where only the first term is non-zero in each of the equations (2) and (3) and assume that 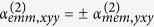, with the rest of the Cartesian components being negligible. According to equations (2) and (3), these terms share the same pathway through the magnetic field of the fundamental beam at *ω*_2_. As a result, switching the phase of *just the magnetic* vector of this fundamental wave according to arrangement (ii) in Fig. 1(b) (keeping the beam at *ω*_1_ in place), will produce a *simultaneous* phase change in *both* the nonlinearly generated electric and magnetic dipolar modes. This results in a *simultaneous* sign change for both terms in equation (1). As a result, the directions of the interferometric suppression and doubling of the nonlinearly generated field will remain the same as in set (i).

On the other hand, if one directs the pump wave at *ω*_2_ according to set (iv) in Fig. 1(b), none of the terms in equation (1) will change their sign: since only the electric vector’s phase will be switched, the resulting phase of the nonlinearly generated dipolar modes will not be affected. Thus, the generation direction, compared to set (i) in Fig. 1(b), again will be preserved. Also, there will be no change in the generation direction with arrangement (iii). In this instance, the opposite (with respect to set (i)) phase of the magnetic vector of the fundamental wave will induce a switch of phase in both the electric and magnetic nonlinearly produced dipolar modes. Meanwhile, the opposite phase of the electric vector will induce no change at all. The result again is the preservation of the direction of nonlinear generation.

According to the above discussion, the direction of nonlinear generation is expected to be preserved with respect to a fixed laboratory coordinate system in all four arrangements shown in Fig. 1(b). Thus, the generation is expected to be non-reciprocal with respect to the direction of the fundamental wave at *ω*_2_. This behavior is qualitatively different from linear scattering, where the pathways are always different for electric and magnetic responses, as it was illustrated in Fig. 1^44^,^49^.

#### Non-reciprocity in the direction of nonlinear generation: general considerations and limitations

A similar non-reciprocity in the nonlinear response will occur for any other combination of effective nonlinear susceptibilities of a similar strength, as long as they share the same pathway, whether electric or magnetic (e.g., the condition 
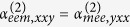
, with the rest of hyperpolarizabilities negligible, ensures the preservation of generation direction when reversing the fundamental wave at *ω*_1_). Non-reciprocity with respect to *all* fundamental waves at once, however, requires the equality of hyperpolarizabilities belonging to different symmetry groups (e.g., 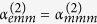), and thus would be forbidden in elements of a single group symmetry. This principle can be extended to higher order nonlinear processes, as well as to higher order multipolar modes (see the example in the following text and the supplementary information for further discussion regarding higher order multipoles).

Note that the non-reciprocity phenomenon described here is not limited to a particular nonlinear process or even to a particular order of the nonlinear response. However, the unidirectional *and nonreciprocal* nonlinear scattering from elements of a single symmetry group is not expected to occur for processes that are degenerate in both the frequency and the direction of the excitation beams, such as harmonic generation with a single fundamental wave. For example, for the second harmonic response, due to the intrinsic permutation symmetry, if either *mme* or *mem* is nonzero, the other term will also be nonzero. As a result, it would be impossible to isolate an effective magnetic hyperpolarizability of only one kind. Similar considerations hold for effective electric hyperpolarizability terms. This is one of the reasons why the phenomenon could not be observed in some previous works that exploited magneto-electric interference in the nonlinearly produced field^30^,^31^,^32^.

#### Non-reciprocity in nonlinear efficiency and inhibition of the nonlinear response

While an option of such non-reciprocity in the direction of nonlinear generation is unique by itself and can be employed in a variety of applications, magneto-electric interference between various effective hyperpolarizability terms can lead to other phenomena that are unavailable with natural nonlinear media. As seen from equations (2) and (3), each mode (electric or magnetic) can, by itself, result from the interference effects between various terms entering the right-hand sides of these equations. Note that, in either the electric or magnetic mode, the terms within a single symmetry group (the terms in each square bracket) depend on a different type of fundamental field vector of a given frequency. Consequently, reversing the direction of one of the fundamental waves will produce a sign change for one of the two interfering terms in each bracket. As a result, the nonlinear dipolar responses will be different for the co- and counter-propagating pumps. In general, all the terms within a single symmetry group on the right hand sides of equations (2) or (3) are expected to be nonzero, at least to some extent, in an arbitrary nanoelement exhibiting a linear magnetic dipolar response (unless a special effort is made to design the nanoelement with only specific dominant effective hyperpolarizability terms). Therefore, when the direction of one or more (but not all) of the excitation beams is reversed or otherwise varied, most of the nanoelements should exhibit a change in the efficiency in the nonlinear response. This expectation is valid even for nanoelements that are entirely symmetric, both in terms of their geometry and material composition. Thus, non-reciprocity with respect to the direction of a subset of fundamental beams in terms of the efficiency of the nonlinear response is inherent to the nonlinear response of most nanoelements, even symmetric ones, and for most nonlinear processes. Furthermore, if a geometry/spectral position can be found that ensures the equality of *all* of the terms within one of the symmetry groups (e.g., 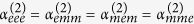, with the rest terms zero), the nonlinear generation will be, according to equation 1, unidirectional for co-propagating pumps, but inhibited for counter-propagating pumps.

In the following section, we consider an example of implementing the proposed general concept with physical structures, and present a numerical study of non-reciprocity in the direction of nonlinear generation.

### Physical implementation: hyperpolarizability retrieval and numerical example

**Figure 2:**
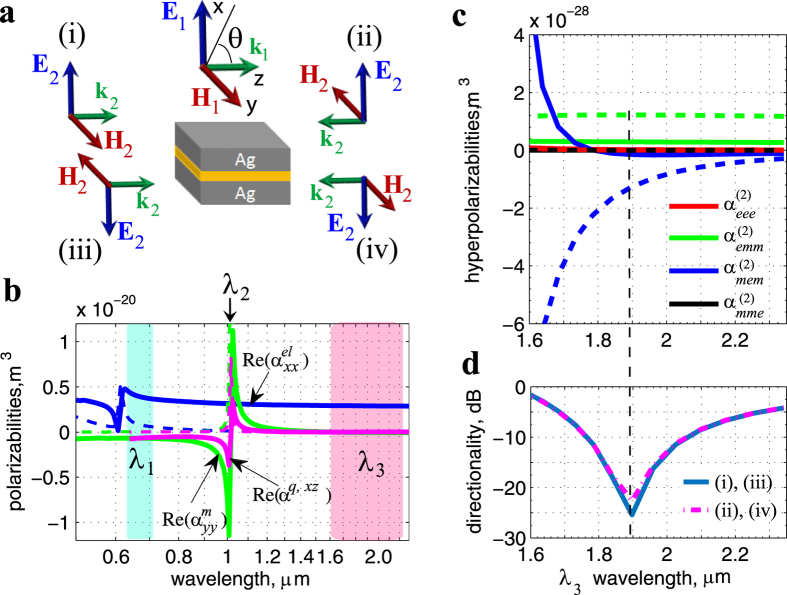
Effective hyperpolarizability retrieval, predicting the optimal spectral position for non-reciprocal nonlinear generation. (**a**) The four pump arrangements used in the analysis in Fig. 2(d) and in Fig. 3. (**b**) Real (*solid*) and imaginary (*dashed*) parts of the retrieved linear electric (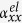) and magnetic (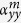) dipolar polarizabilities, and the electric quadrupolar (*α*^*q*,*xz*^) polarizability; *λ*_1_ and *λ*_2_ indicate the wavelengths used to produce the DFG response at *λ*_3_ in (**c**,**d**). (**c**) Retrieved real (*solid*) and imaginary (*dashed*) parts of the effective hyperpolarizabilities. (**d**) Directionality *D* attains peak in the negative *z* direction at 1.9 *μ*m, where 
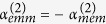
 and the rest of the hyperpolarizabilities are negligible, for all four configurations shown in (**a**). A strongly directional and non-reciprocal, with respect to the pump at *ω*_2_, nonlinear generation is observed. A small difference between the forward ((i),(iii)) and reverse ((ii), (iv)) excitations is due to the nonzero 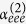 term, which does not share the same pathway through the magnetic field of the wave at *ω*_2_ with the 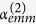 and 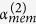 terms; hence, it has a different sign when the pump direction is reversed, modifying the resulting electric and magnetic dipolar modes (equations (2) and (3)).

To verify the feasibility of observing the above phenomena in physical nanoelements, we considered the process of difference frequency generation (DFG) in plasmonic dimers, formed by two silver strips separated by a thin layer of dielectric (Fig. 2(a)). We chose the nonlinear process to ensure that the nanoelement would be small compared to the nonlinearly generated wavelength, thus allowing us to minimize the manifestation of higher order multipoles in the nonlinearly generated field. Our preliminary hydrodynamic model^50^,^51^,^52^ analysis has shown that the main contribution to the second-order nonlinear response in such a geometry results from the nonlinearity of the spacer material. This determination is in line with recent results on the third order response in similar structures^53^. The nonlinear response due to the metal has been thus neglected in further analysis. Furthermore, the simulations have shown that the major contribution to the nonlinear response comes from the 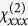 component of the intrinsic second-order nonlinear susceptibility tensor; other tensor components produce a negligible impact, even though accounted for. This result is expected, due to the dominance (up to two orders of magnitude) of the local fundamental electric field component orthogonal to the spacer layer within the spacer volume^44^,^49^. Thus, we chose the 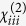 tensor component of the BBO spacer to be along the *x* axis.

Linear polarizabilities for the dimer geometry from Fig. 2(a), retrieved using our previously developed procedure^49^, are shown in Fig. 2(b). The same procedure can be used to identify the dipolar and quadrupolar partial waves in the nonlinearly generated field. One can repeat the procedure for four combinations of fundamental beam directions, arriving at the set of expressions for the effective nonlinear polarizabilities of the following form (see supplemental materials for the derivation details):


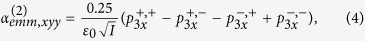



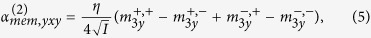


with similar expressions holding for the rest of the eight hyperpolarizabilities entering equations (2) and (3) (see supplemental information). In equations (4) and (5), the 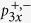 (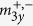) and similar terms indicate the amplitude of the *x* (*y*) component of the electric (magnetic) dipolar mode generated at frequency *ω*_3_ and retrieved for a certain combination of forward (“+”) and reverse (“−”) directions of the fundamental beams. The first (second) superscript refers to the direction of the wave at *ω*_1_ (*ω*_2_). Figure 2(c) presents the resulting hyperpolarizabilities when the frequency *ω*_2_ is placed at the magnetic resonance peak, while sweeping *ω*_1_ (Fig. 2(b). We verified the accuracy of both linear and nonlinear retrievals by ensuring a very good agreement between the numerical and the analytical far field angular distributions (to obtain the latter, we used the retrieved dipolar and quadrupolar amplitudes) (see supplemental information for details). Note that, although all axial hyperpolarizabilities were allowed in the retrieval, they vanished nearly identically, indicating the predominant polar nature of the dimer geometry.

Because the spectral location of the DFG response was far below the frequencies of both the electric and magnetic resonances of the nanoelement, the dipolar modes contributed an overwhelming majority (above 95%; see Fig. S5 in supplementary information for the quantitative data) of the nonlinearly generated field. This result was expected, and it justifies giving no consideration of higher order multipoles in the nonlinear response in Fig. 2(c). According to Fig. 2(b), there is also a negligible contribution from modes other than the electric dipolar mode for the field at *ω*_1_ (see also Fig. S3 in supplementary information). However, the electric quadrupole response accounts for about 20% of the fundamental field at *ω*_2_. Hence, from that fundamental field, there is some quadrupole contribution into both the “*emm*” and the “*mem*” nonlinear dipolar terms (e.g., the 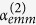 term is, strictly speaking, 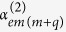, where *q* indicates the electric quadrupole contribution of the fundamental field at *ω*_2_ to the nonlinearly produced electric dipolar mode). Assuming the linearly polarized plane wave excitation considered in the present study, however, the phase of the fundamental electric quadrupolar contribution (the latter proportional to the symmetric part of the gradient of the electric field^54^) changes in a way similar to that of the magnetic dipolar mode of the same field, for all four orientations of the fundamental field vectors shown in Fig. 2(a) (see supplementary information for a more detailed description). That similarity allowed us to use a single effective hyperpolarizability for each of the *emm* and *mem* terms, rather than accounting separately for the magnetic dipolar and electric quadrupolar contributions into the nonlinearly generated dipolar modes. Both the dipolar and the quadrupolar inputs of the fundamental field were, however, accounted for in the retrieved hyperpolarizabilities.

## Discussion

As seen in Fig. 2(c), 
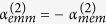
, and the remaining hyperpolarizabilities are either identically zero or negligible near 1.9 *μ*m, reproducing the situation discussed earlier. Therefore, when the beam at *ω*_1_ is propagating in the positive *z* direction (Fig. 2(a)), one can expect the nonlinear generation near 1.9 *μ*m to be interferometrically suppressed in a hemisphere encompassing the negative *z* semi-axis, independently of the (forward or reverse) direction of the beam at *ω*_2_.

**Figure 3:**
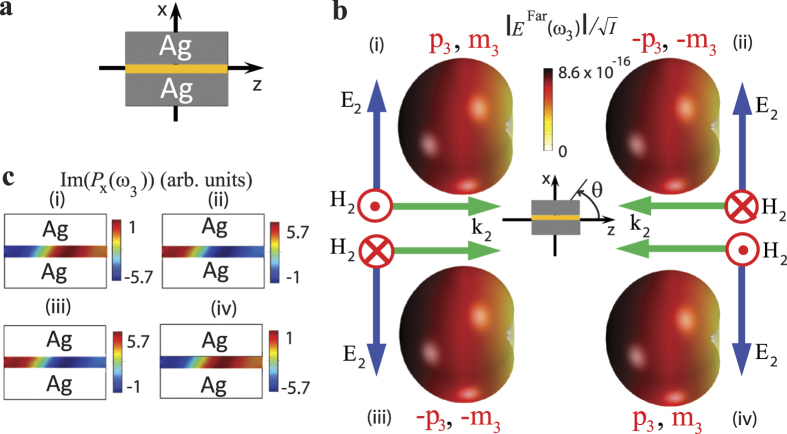
Demonstration of directional and non-reciprocal nonlinear generation, where the direction of the nonlinearly produced field is decoupled from that of the fundamental beam at *ω*_2_, and the corresponding induced intrinsic nonlinear polarization. (**a**) Geometry cross section used in (**c**). (**b**) Angular distribution of the nonlinearly generated far field for the four pump arrangements from Fig. 2(a) at *λ*_3_ = 1.9 *μ*m. The only non-negligible hyperpolarizabilities are 
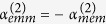
 at *λ*_3_ = 1.9 *μ*m. Because the same “pathway” is shared through the magnetic field of the fundamental wave at *ω*_2_ (equations (2) and (3)), both (nonlinearly induced) electric and magnetic dipolar modes (indicated p_3_ and m_3_) change their sign *simultaneously* when the direction of the magnetic field vector of that fundamental wave is reversed, compared to (i) (sets (ii) and (iii)); while no change occurs in (iv), compared to (i). According to equation (1), the direction of the nonlinear generation is preserved in all four arrangements as a result. Some difference between the forward ((i) and (iii)) and the reverse ((ii) and (iv)) excitations is due to the small nonzero 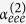 term. (**c**) Dominant component of the nonlinear polarization within the spacer layer at 1.9 *μ*m, for the same four pump arrangements. Although the direction of the nonlinear generation is preserved with all four arrangements, the induced intrinsic nonlinear polarization has the opposite sign in sets (ii) and (iii), with respect to sets (i) and (iv), following the phase of the *magnetic* vector of the fundamental wave at *ω*_2_. This result occurs even though *purely electric intrinsic* second-order susceptibility is assumed. The location of the Ag stripes is shown schematically for clarity.

The analysis of the nonlinearly generated far field confirms the above prediction precisely (Figs. 2(d) and 3(b)), with the directionality ratio 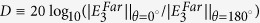 attaining the (negative) 26 dB peak at 1.9 *μ*m for all four pump arrangements shown Fig. 2(a).

Note that one of the pumps (*λ*_2_ in Fig. 2(b)) is placed nearly at the magnetic resonance peak, where the real part of the linear magnetic dipolar (and the real part of the electric quadrupolar) polarizability almost vanishes. Consequently, the real parts of the hyperpolarizabilities are negligible near 1.9 *μ*m. Matching their imaginary parts ensures the unidirectionality of the nonlinear generation. While matching the imaginary parts with the opposite signs is not surprising, due to the presence of (nonlinear) gain, it is worth noting that the limitation on backscattering imposed by the optical theorem can be thus lifted in the process of nonlinear generation, similar to the linear response case in the presence of gain^26^. (Indeed, as seen from equation (1), a perfect backscattering requires matching electric and magnetic dipolar moments with the opposite signs. This requirement makes perfect backscattering impossible for passive elements for which the imaginary parts of multipolar contributions always stay positive. The presence of gain lifts this limitation).

Additionally, we find that, for the dimer geometry, the generation direction is overall independent from the direction of the fundamental beam at *ω*_2_ within the *xz* plane, except for some variation in the nonlinear efficiency for excitation at an angle with respect to the *z* axis. The latter independence can be seen as a result of the dominance of the local fundamental electric field component normal to the spacer layer, for all dimer orientations within the *xz* plane^44^. As a result, a similar non-reciprocity can be expected when using not only plane wave, as in the present example, but also line current sources.

Figure 3(c) shows the *x* component of the nonlinear polarization 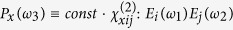 (*i*, *j* standing for *x*, *y*, *z*), induced at 1.9 *μ*m. Because of the placement of *ω*_2_ at the magnetic resonance, where the electric dipolar mode is weak, the phase of the majority of the *local* fundamental electric field *E*_*j*_(*ω*_2_) and, hence, the sign of the nonlinear polarization, follow that of the *magnetic* vector of the incident field of the pump wave at *ω*_2_. As a result, the induced intrinsic nonlinear polarization is of the opposite phase in sets (ii) and (iii), with respect to the phase in sets (i) and (iv). This, in turn, transfers to the *simultaneously* negative phases of the nonlinearly induced electric and magnetic dipolar modes (both related to the volume integral of the intrinsic polarization^54^,^55^) in sets (ii) or (iii), and thus, as discussed earlier, to the preserved direction of energy generation in all sets, from (i) to (iv). This situation is similar to the “negative index” media, where the direction of energy propagation is preserved when the phase velocities of both the electric and the magnetic partial waves are reversed simultaneously^45^,^46^. In the “negative index” scenario the simultaneous phase reversal requires, however, the simultaneously negative values of *both* the permittivity and permeability. In contrast, here, the simultaneous phase reversal of both electric and magnetic vectors of the nonlinearly generated field is achieved through a nonlinear interaction, owing to the flipped phase of *a single* vector of the *fundamental* excitation field (the magnetic one). Such simultaneous reversal is feasible due to the existence of the shared pathways inducing the electric and magnetic nonlinear responses through the magnetic field of the wave at *ω*_2_. These common pathways, in turn, arise owing to the multiplicative nature of the nonlinear response. Note that the nonlinear case is also in contrast with the linear response discussed in relation to set (iii) of Fig. 1(b) (where the simultaneous phase reversal is due to the flipped phases of both electric and magnetic vectors of the excitation field).

In conclusion, we have shown that the involvement of multiplicative processes in nonlinear multipole interference allows us to achieve directional and non-reciprocal (with respect to the direction of one or several pump beams) nonlinear generation from subwavelength objects. We developed the procedure for the retrieval of the effective nonlinear magneto-electric polarizabilities and demonstrated numerically that the described phenomena can be achieved in the optical frequency range, using purely symmetric dimer structures (both in terms of their geometry and material composition) with realistic materials parameters. The geometry is not unique and can be optimized further for fabrication purposes. We also predicted that interference between various hyperpolarizability terms *within* the nonlinearly generated multipolar modes would inhibit a certain nonlinear process in a subwavelength unit designed to have the same strength of all symmetry-allowed types of its hyperpolarizabilities; while reversing the direction of one of the pumps would switch it back on. This latter phenomenon might be especially useful in the radio frequency range, where a nonlinear response can be strong, but is often undesirable in applications. In addition to the manifestation of multipolar responses of opposite parities but comparable strengths, both non-reciprocal directionality and inhibition of the nonlinear response require a *certain relation* between various hyperpolarizability terms *within each* (nonlinearly generated) multipolar mode itself. This relationship and these phenomena are not expected to be available in natural nonlinear media, but they can be achieved by the design of effective nonlinear multipolar response of structured materials. The proposed concept offers the potential of achieving all the presented phenomena via the response of sub-wavelength elements, thus allowing their use as building blocks in developing a nonlinear media with similar unique features. Combined with strong enhancements of the nonlinear response offered by plasmonic metasurfaces, the suggested approach can allow one to engineer artificial nonlinear nanomaterials with an enhanced and inherently non-reciprocal response. Such a capability can serve as a keystone in a variety of applications in integrated optics and photonics.

## Methods

Finite elements method incorporated in COMSOL Multiphysics software package (www.comsol.com) has been used for the numerical analysis. Air is assumed for the surrounding medium. The dimer geometry consists of 120 × 120 × 35 nm silver strips separated by the 11 nm thick BBO spacer. The permittivity of silver and BBO follow the data in^56^,^57^, respectively. Intrinsic nonlinear hyperpolarizability of BBO is according to the data in ref. 58 (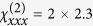 pm/V). The previously developed procedures which have shown to produce an exact agreement with analytical results and a very good agreement with experimental data have been used for simulations of the nonlinear response^43^ and for the hydrodynamic model analysis^51^,^52^.

## Additional Information

**How to cite this article**: Poutrina, E. and Urbas, A. Multipolar interference for non-reciprocal nonlinear generation. *Sci. Rep.* **6**, 25113; doi: 10.1038/srep25113 (2016).

## Supplementary Material

Supplementary Information
