# Supplementary material for: Multipolar interference for non-reciprocal nonlinear generation
Source: Sci Rep. 2016 Apr 29;6:25113. doi: 10.1038/srep25113 (PMC4850388; doi:10.1038/srep25113)
Supplement: Supplementary Information [file srep25113-s1.pdf]

Supplementary Information for "Multipolar interference for non-reciprocal nonlinear generation"

Ekaterina Poutrina[1,2,*] **and Augustine Urbas**[1]

[1]Materials and Manufacturing Directorate, Air Force Research Laboratory, Wright Patterson Air Force Base, Ohio 45433, USA
[2]UES, Inc., 4401 Dayto-Xenia Road, Dayton, OH 45432, USA
[*]ekaterina.poutrina.ctr.ru@us.af.mil

Linear polarizabilities retrieval

The orientation of the dimer structure with respect to the incident field used for the retrieval of linear polarizabilities in Fig. 1(b) and in simulations presented here is shown in Fig. S1.

Linear polarizabilities shown in Fig. 1(b) are retrieved following the procedure developed in Ref. [S1]. A brief summary is outlined below. The "xx" component of electric dipolar (α^e_{xx}), "yy" component of magnetic dipolar (α^m_{yy}), and the electric quadrupolar polarizability responsible for inducing the "xz" component of electric quadrupolar tensor ($\alpha^{q,xz}$) are found by probing the amplitude A of the scattered far field in the three orthogonal directions as following:

$$\alpha^{el}_{xx} = \frac{4\pi}{k^2} A|_{\substack{\theta=90^\circ \\ \varphi=90^\circ}}, \tag{S1a}$$

$$\alpha^{m}_{yy} = \frac{4\pi}{k^2} A|_{\substack{\theta=90^\circ \\ \varphi=0^\circ}}, \tag{S1b}$$

$$\alpha^{q,xz} = \frac{4\pi}{k^2} A^q|_{\theta=0^\circ}, \tag{S1c}$$

In the above expressions, A is related to the scattered electric far field $\mathbf{E}^{sc}_{far}$ as

$$\begin{aligned}\mathbf{E}^{sc}_{far} &\equiv \frac{e^{ikr}}{r}\mathbf{A}E_0 \\ &= \frac{e^{ikr}}{r}\left(\mathbf{A^{el}} + \mathbf{A^m} + \mathbf{A^q}\right)E_0,\end{aligned} \tag{S2}$$

where $\mathbf{A}^{el}$, $\mathbf{A}^m$, and $\mathbf{A}^q$ denote the amplitudes of the electric dipolar, magnetic dipolar, and the electric quadrupolar partial waves, respectively (bold font indicates vector quantities), $E_0 = E_{x0}$ is the amplitude of the incident field, and linear dipolar polarizabilities relate the incident electric ($\mathbf{E}_0 = (E_{x0}, 0, 0)^{\mathrm{T}}$) and magnetic ($\mathbf{H}_0 = (0, E_{x0}/\eta, 0)^{\mathrm{T}}$) fields to the induced electric and magnetic dipolar moments as $\mathbf{p}_0 = \varepsilon_0 \overline{\overline{\alpha^{el}}}\mathbf{E}_0$ and $\mathbf{m}_0 = \overline{\overline{\alpha^m}}\mathbf{H}_0$. Note that the normalization for the quadrupolar amplitude $\mathbf{A}^q$ is chosen such that it enters Eq. (S2) as an addition to the magnetic and electric dipolar modes, allowing to factorize the incident field amplitude (see Ref. [S1] for the details).

The far field scattering amplitude $A(\hat{\mathbf{r}}_0, \omega)$ in a given direction $\hat{\mathbf{r}}_0$ ($\hat{\mathbf{r}}_0$ being a unit vector) is found numerically by integrating the scattered electric and magnetic near fields over an arbitrary surface enclosing the dimer structure using the Stratton-Chu formula [S1,S2]. The angular distributions for the three multipole modes entering Eq. (S2) (assuming the dipolar modes are along the incident field vectors, $\mathbf{p}_0 = (p_{x0}, 0, 0)^{\mathrm{T}}$, $\mathbf{m}_0 = (0, m_{y0}, 0)^{\mathrm{T}}$) are visualized in Fig. S1(a). As shown in ref. S1 and is seen from the figure, with Q^{xz} being the only nonzero quadrupolar tensor component and with no higher than the quadrupolar terms, scattering into the xy plane comes only from the dipolar modes. Due to their orthogonality, the dipolar modes are decoupled in the xy plane such that $A^{el}|_{\theta=90^\circ} = A_x|_{\theta=90^\circ}$ and $A^m|_{\theta=90^\circ} = A_z|_{\theta=90^\circ}$. Using the regular relations for the angular dependence of the far field amplitude of oscillating electric and magnetic dipoles ($\mathbf{A}^{el} \sim \mathbf{r}\times\mathbf{r}\times\mathbf{p}_0/E_0$, $\mathbf{A}^m \sim \mathbf{r}\times\mathbf{m}_0)/H_0$), dipolar amplitudes are found by probing the scattered far field in the two orthogonal directions along the x and y axes according

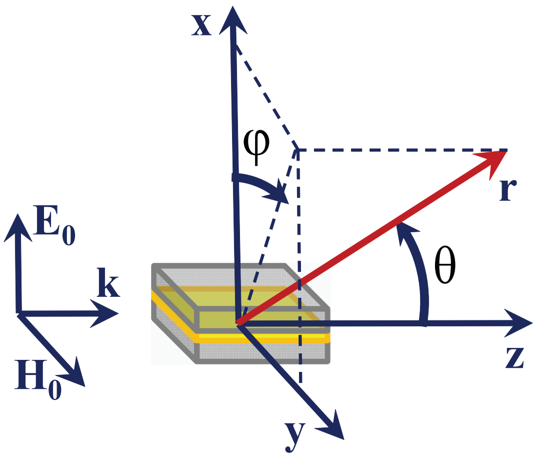

Figure S1. Dimer orientation with respect to the Cartesian and Spherical coordinates and with respect to the incident field used in the linear polarizabilities retrieval.

to:

$$p_{x0} = \frac{4\pi\varepsilon_0 E_0}{k^2} A|_{\substack{\theta=90^\circ \\ \varphi=90^\circ}}, \tag{S3a}$$

$$m_{y0} = \frac{4\pi E_0}{\eta k^2} A|_{\substack{\theta=90^\circ \\ \varphi=0^\circ}}, \tag{S3b}$$

from which Eqs. (S1a) and (S1b) follow (note that the use of the total amplitude A, rather than of its x and z components, is not a mistake in Eq. (S3), due to the orthogonality of the two Cartesian components [S1]). The amplitude $\mathbf{A}^q$ of the electric quadrupolar contribution is subsequently found as the difference between the numerical value of the amplitude scattered in the forward $(\theta = 0)$ direction and the one that would have resulted from the sum of the retrieved dipolar contributions alone, $A^q|_{\theta=0^\circ} = A|_{\theta=0^\circ} - \left[A|_{\substack{\theta=90^\circ \\ \varphi=90^\circ}} + A|_{\substack{\theta=90^\circ \\ \varphi=0^\circ}}\right]$.

The retrieved dipolar and quadrupolar amplitudes are then used in the following analytic expressions for the angular distributions of their various Cartesian components (see Ref. [S1] for the derivation), for comparison with the numerical results:

$$A_x^{xy} = A|_{\substack{\theta=90^\circ \\ \varphi=90^\circ}} \sin^2(\varphi), \tag{S4a}$$

$$A_z^{xy} = A|_{\substack{\theta=90^\circ \\ \varphi=0^\circ}} \cos(\varphi), \tag{S4b}$$

$$A_x^{q,xz} = A^q|_{\theta=0^\circ} \cos^3(\theta), \tag{S4c}$$

$$A_z^{q,xz} = A^q|_{\theta=0^\circ} \sin(\theta)\cos^2(\theta). \tag{S4d}$$

Note that, according to Eqs. (S3), (S4a), and (S4b), the x- and z-components of the scattering amplitude taken in the xy plane represent purely electric and magnetic dipolar modes, respectively.

Examples of such a comparison for the spectral positions used to produce the DFG response of Fig. 2 are shown in Figs. S2 and S3. An exact agreement is seen for all curves at the "magnetic" resonance position at 1 μm (Fig. S2). The agreement is also exact for the dipolar modes in all cases considered (Figs. S2(b) and S3(a)), and there is less than 3% discrepancy for the combined scattering amplitude at 662 nm (Fig. S3(c)). Some discrepancy seen for the norm of the quadrupolar mode at 662 nm (Fig. S3(b)) is due to the manifestation of higher order multipoles at this frequency range which is noticeably above the "magnetic" (lowest in frequency) resonance. The qualitative shape of the quadrupolar angular distribution, however, agrees well for all field components, and noting that the quadrupole mode by itself constitutes a small fraction of the scattering response at this frequency range, it results in less than the 3% discrepancy in the total scattering response (Fig. S3(c)); we therefore did not pursue a higher order retrieval. As discussed in Ref. [S1], in addition to demonstrating the accuracy of the retrieval, the agreement between the analytical and numerical angular distributions validates the assumption of the lack of dipolar components in the directions other than those along the incident field vectors being induced in the nanoelement (no optical activity exhibited by the nanoelement), as well as the assumption of no multipoles of an order higher than the electric quadrupolar mode being significant.

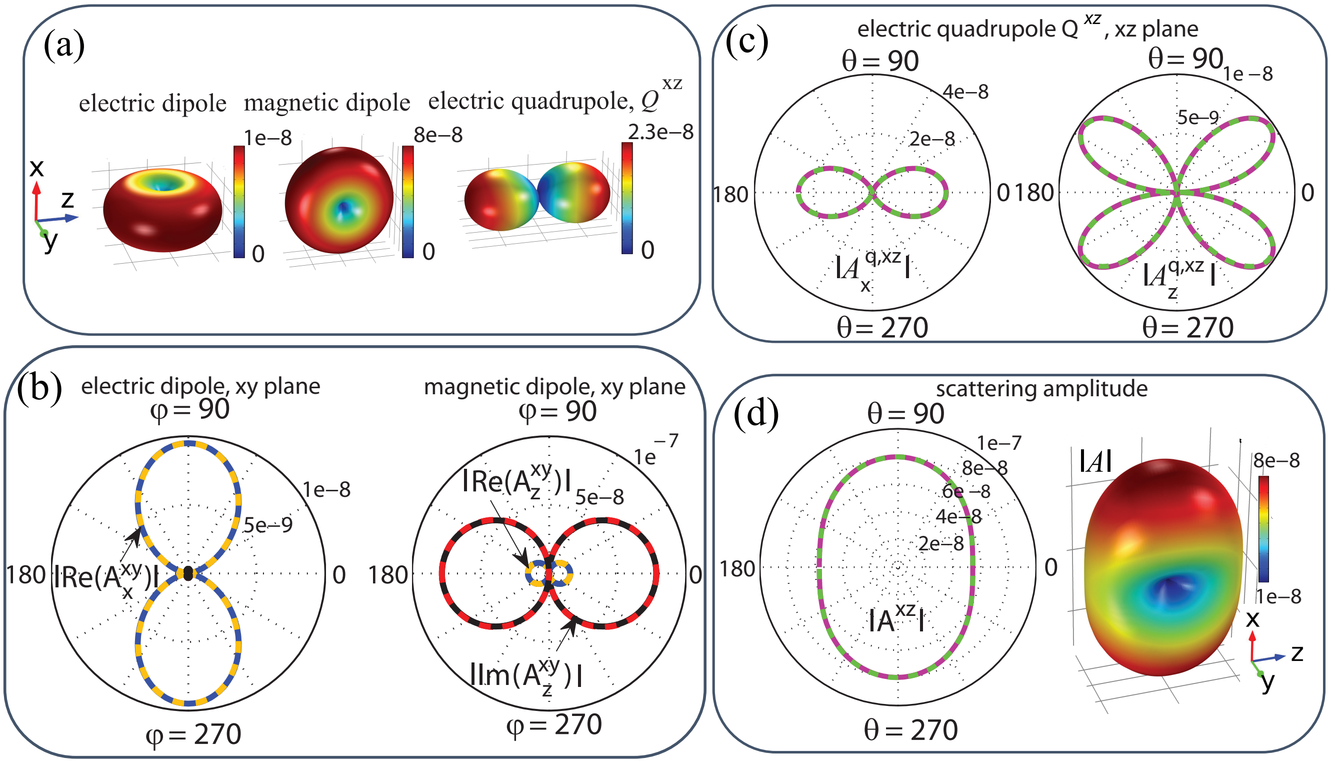

Figure S2. (a) Schematic illustration of the angular distribution of the far field amplitudes for the dipolar and the electric quadrupolar multipole components induced via linear response in the dimer geometry with the excitation as in Fig. S1. (b)-(c): Analytical (*solid*) versus numerical (*dashed*) angular distributions of (b) the dipolar, (c) the electric quadrupolar, and (d) the combined far-filed scattering amplitude at 1 μm ("magnetic" resonance, λ_2 in Fig. 1(b)), showing an exact agreement between the numerical and the analytical results in all cases. (b) *Solid blue* and *solid red* lines show numerical values of the real and the imaginary parts, respectively, of the A_x and A_z components of the scattered far field in the *xy* plane. *Dashed yellow* and *dashed black* show analytical values of the real and the imaginary parts, respectively, of the electric and magnetic dipolar modes calculated using Eqs. (S4a) and (S4b), which assume the induced dipoles are along the incident field vectors. The exact agreement between the numerical and the analytical curves validates the proposition that no other multipoles scattering into the *xy* plane. Note that in this case the *x*- and *z*-components of the scattering amplitude taken in the *xy* plane represent purely electric and magnetic dipolar modes, respectively. (c) *Solid Purple*: the numerical value of the norm of the quadrupole far field amplitude in the *xz* plane found as the difference between the numerical distribution of the scattered far field in the *xz* plane and the prediction based on the dipolar contributions alone. *Dashed green*: analytical prediction for that difference, calculated using Eqs. (S4c) and (S4d). (d) The *solid purple*: the numerical far-field amplitude scattered into the *xz* plane. *Dashed green*: analytical prediction found by combining the dipolar and the electric quadrupolar modes, in exact agreement with the numerical result.

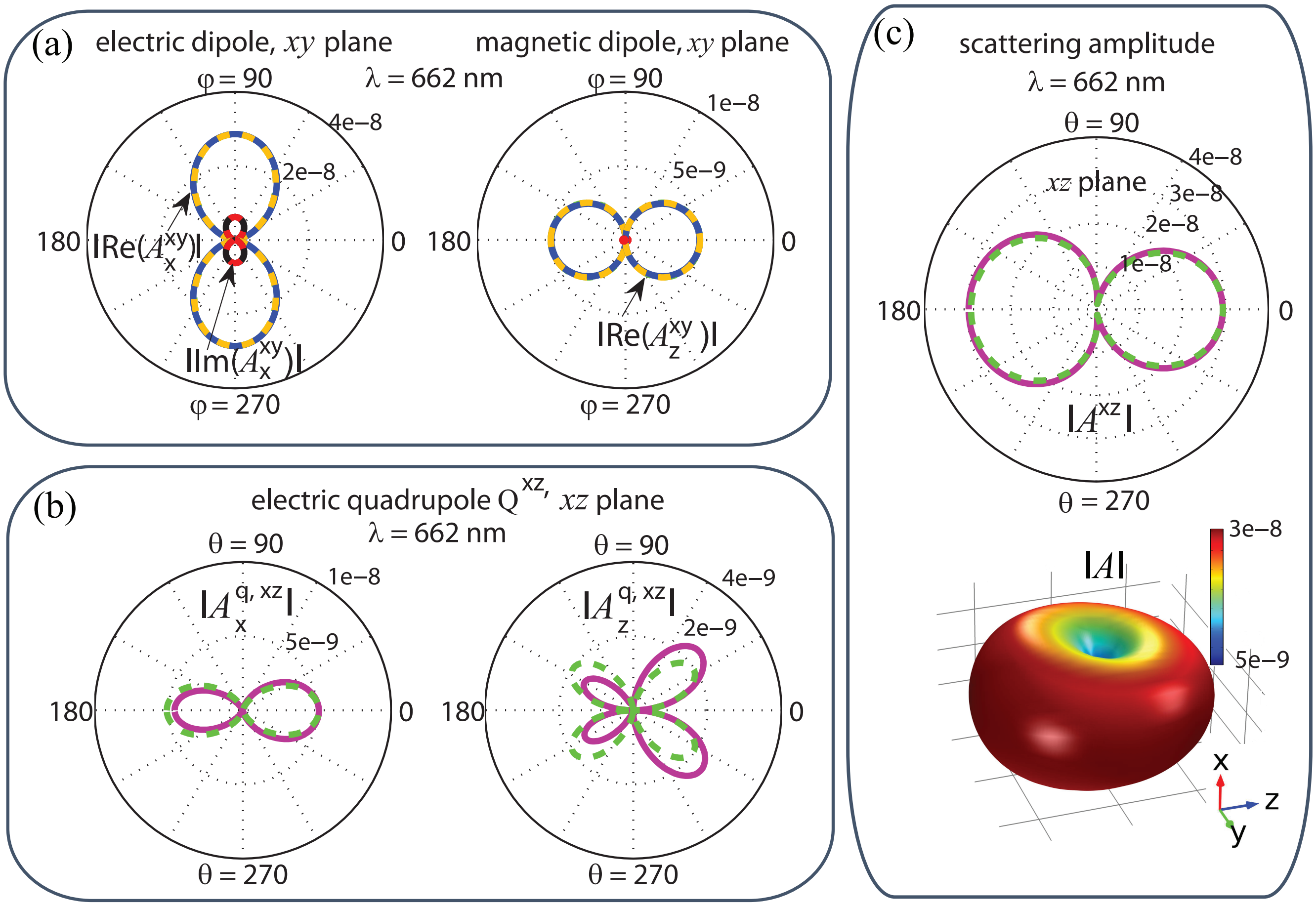

Figure S3. Comparison of numerical (*solid*) and analytical (*dashed*) results similar to that in Figs. 2(b)-2(c), but calculated at 662 nm–the λ_1 wavelength used to produce the DFG response in Fig. 2 in the main text. The observed agreement is again exact for the dipolar terms (panel (a)) and there is a less than 3% discrepancy between the analytical and numerical results in the total scattering response (panel (c), top). A small discrepancy in the electric quadrupolar response (panel (b)) is due to the presence of higher order multipoles. Since at that frequency range the quadrupolar mode by itself constitutes a small fraction of the total response, the difference results in less than 3% discrepancy in the total scattering response (panel (c)).

A note on variation of linear quadrupole contribution when reversing the direction of the excitation beam

As seen in Figs. 2(b) and as shown in the preceding section, while quadrupole contribution is negligible for the whole range of wavelengths λ_1 used in the present study (blue region in Fig. 2(b)), electric quadrupole contribution constitutes about 20% of the fundamental scattered field at λ_2 (Figs. 2(b) and S2). As a result, both the $\alpha^{(2)}_{emm}$ and the $\alpha^{(2)}_{mem}$ terms in the nonlinearly induced dipolar modes arise owing to both the magnetic dipolar and the electric quadrupolar partial waves of that fundamental field. However, as we show below, assuming linearly polarized plane wave excitation considered in the present study, the phase of the fundamental electric quadrupolar contribution follows the same pattern as that of the magnetic dipolar mode of the same field, for all four orientations of the fundamental field vectors shown in Fig. 2(a). That allowed us to use a single effective hyperpolarizability for each of the "emm" and "mem" terms, rather than accounting separately for the magnetic dipolar and quadrupolar contributions into the nonlinearly generated dipolar modes, as explained below.

As shown in ref. 49, the quadrupolar source producing the xz component of the electric quadrupolar response shown in the previous section can be seen as formed by two x-oriented, closely spaced along z direction, and oscillating out-of-phase electric dipolar modes, with its electric field given by

$$\mathbf{E^q} \quad = \tilde{\mathbf{E}}^{el}(0)\, e^{-i\mathbf{k}\cdot\mathbf{a}} + \tilde{E}^{el}(0)\, e^{i\delta} e^{+i\mathbf{k}\cdot\mathbf{a}}. \tag{S5}$$

In the above equation, the two dipolar modes are assumed shifted small distances $\pm a$ from the origin along the propagation direction (z), $ka \leq 2\pi$, $\tilde{\mathbf{E}}^{el}(0)$ is the amplitude of each dipolar mode when placed at the origin, and $\delta = \pi$ is the relative phase between the two modes. For the four orientations of the excitation wave shown in sets (i)-(iv) in either Fig. 1(b) or 2(a), the dot product $\mathbf{k}\cdot\mathbf{a} = ka\cos\theta$ in the exponent in Eq. S5 transforms as following: (i) ka; (ii) $-ka$; (iii) ka; (iv) $-ka$. The induced phase of oscillations of the dipolar amplitude $\tilde{\mathbf{E}}^{el}(0)$, on the other hand, follows that of the inducing electric vector. Denoting $\tilde{\mathbf{E}}^{el}(0)$ as the amplitude induced in set (i), the amplitude and phase in the rest of the arrangements are as following: (ii) $\tilde{\mathbf{E}}^{el}(0)$; (iii) $-\tilde{\mathbf{E}}^{el}(0)$; (iv) $-\tilde{\mathbf{E}}^{el}(0)$. Using these values in Eq. S5 and following the steps from Ref. 49 for each of the (i)-(iv) arrangements, one arrives at the following expressions for the field of the quadrupole partial wave scattered linearly by the dimer structure, with each of the four pump orientations:

$$\text{(i)} \quad \mathbf{E}^q = -\tilde{\mathbf{E}}^{el}(0)\, 2ika\cos\theta; \tag{S6a}$$
$$\text{(ii)} \quad \mathbf{E}^q = \tilde{\mathbf{E}}^{el}(0)\, 2ika\cos\theta; \tag{S6b}$$
$$\text{(iii)} \quad \mathbf{E}^q = \tilde{\mathbf{E}}^{el}(0)\, 2ika\cos\theta; \tag{S6c}$$
$$\text{(iv)} \quad \mathbf{E}^q = -\tilde{\mathbf{E}}^{el}(0)\, 2ika\cos\theta; \tag{S6d}$$

As seen from Eq. S6, in each of the four arrangements, the phase of the resulting electric quadrupole scattered field pattern changes in a similar fashion as that of the magnetic vector of the incident wave shown in Fig. 2(b). Since the latter defines the phase of the induced magnetic dipolar moment, the quadrupolar and the dipolar partial waves change phase in a similar way when reversing the direction of the linearly polarized incident plane wave using any of the (i)-(iv) scenarios and thus always contribute in-phase to the nonlinear response.

While a complete analysis of quadrupolar response with an arbitrary excitation waveform is beyond the present study, one can arrive at the above result for the linearly polarized plane wave excitation in a more general way noting that the quadrupolar moment interacts with the symmetric part of the gradient of the excitation field and thus its partial wave can be expressed as:[3,4]

$$\mathbf{E}^q \sim q^{el}\,\frac{\nabla\mathbf{E}_0 + (\nabla\mathbf{E}_0)^{\mathrm{T}}}{2}, \tag{S7}$$

where q^{el} formally denotes the quadrupolarizability of the nanoelement. Assuming the linearly polarized plane wave excitation, the amplitude of the incident wave at ω_2 is given as following for the four pump orientations of Fig. 2(a): (i) $\mathbf{E}_0 = E_0\hat{x}\exp(ikz)$; (ii) $\mathbf{E}_0 = E_0\hat{x}\exp(-ikz)$; (iii) $\mathbf{E}_0 = -E_0\hat{x}\exp(ikz)$; (iv) $\mathbf{E}_0 = -E_0\hat{x}\exp(-ikz)$. Using these expressions in Eq. S7, one arrives at the result similar to the one of Eq. S6 where the sign in the sets (ii) and (iii) is opposite of that in (i) and (iv), thus varying in a similar fashion as that of the induced magnetic dipolar mode. The simultaneous phase change of the two modes is also confirmed in numerical simulations.

Since both electric quadrupolar and magnetic dipolar partial waves also share the same (odd in electric field) parity, we do not aim at distinguishing between the contributions of these partial waves into the *nonlinearly* produced dipolar modes and use the notation $\alpha^{(2)}_{emm}$ and $\alpha^{(2)}_{mem}$ (rather than $\alpha^{(2)}_{em(m+q)}$ and $\alpha^{(2)}_{me(m+q)}$, where "q" would stand for the quadrupolar fundamental partial wave) for the effective hyperpolarizabilities. The retrieved hyperpolarizabilities thus, however, do account for both the quadrupolar and the dipolar inputs of the fundamental field at λ_2 into the nonlinear response.

Nonlinear polarizabilities retrieval

A similar approach can be used for analyzing the multipolar components in the nonlinearly generated field. Analysis of the nonlinearly generated far field has shown that the dipolar components similarly de-couple in the xy plane and, for the dimer orientation shown in Fig. S1, are aligned along the incident field vectors. Comparison of the analytical and numerical angular distributions for the multipolar components produced by DFG process at $\lambda_3 = 1.9\ \mu$m with the co-directional excitation (Fig. S4(a)) is shown in Fig. S5, demonstrating an exact agreement for the dipolar modes and an excellent (less than 0.25% discrepancy) agreement for the combined scattering amplitude. Eq. (S4) is used for calculating the analytical curves, with A, in this case, being the amplitude of the nonlinearly generated field. Note that, as expected, the use of the DFG process allowed to minimize the contribution from higher order modes in the nonlinearly generated field: the dipolar modes shown in Fig. S5(a) constitute more than 95% of the total nonlinearly generated field (Fig. S5(c)).

As discussed in the main text, the sign with which various terms enter the right hand side of Eqs. (2) and (3) changes when switching the phase of the fundamental electric or magnetic field inducing the corresponding term. The latter phase change occurs for magnetic (electric) field when reversing the propagation direction of the incident beam by 180° rotation around the x (y) axis, as illustrated by sets "(ii)" ("(iv)") in Fig. 2 in the main text, and for both electric and magnetic fields with the 180° rotation around the z axis (set "(iii)" in Fig. 2). To retrieve all eight terms of the effective hyperpolarizabilities entering Eqs. (2) and (3), one can then repeat the retrieval procedure similar to the one used in Fig. S5 for four different pump arrangements, alternating the direction of each of the fundamental beams between the two opposite excitations–e.g., by choosing the four sets shown in Fig. S5–and proceeding as described below.

Denoting by "$+\mathbf{E}_0$" and "$+\mathbf{H}_0$" the incident fields for fundamental beams at both ω_1 and ω_2 in Fig. S4(a) (configuration "+,+"), the incident fundamental fields in the rest of the arrangements in Fig. S4, written in terms of $\mathbf{E}_0$ and $\mathbf{H}_0$, appear as following: $+\mathbf{E}_0(\omega_1)$, $+\mathbf{H}_0(\omega_1)$, $+\mathbf{E}_0(\omega_2)$, $-\mathbf{H}_0(\omega_2)$ for configuration "+,–"; $+\mathbf{E}_0(\omega_1)$, $-\mathbf{H}_0(\omega_1)$, $+\mathbf{E}_0(\omega_2)$, $+\mathbf{H}_0(\omega_2)$ for configuration "–,+"; and $+\mathbf{E}_0(\omega_1)$, $-\mathbf{H}_0(\omega_1)$, $+\mathbf{E}_0(\omega_2)$, $-\mathbf{H}_0(\omega_2)$ for configuration "–,–". Using this notation in Eqs. (2) and (3) for each of the four arrangements shown in Fig. S4, one arrives at the following sets of equations for the electric and magnetic dipolar modes:

$$\mathbf{p_3}^{+,+} = \frac{\varepsilon_0}{\sqrt{I}}\left[\alpha^{(2)}_{eee} : \mathbf{E_{01}E_{02}} + \eta^2\alpha^{(2)}_{emm} : \mathbf{H_{01}H_{02}} + \eta\,\alpha^{(2)}_{eem} : \mathbf{E_{01}H_{02}} + \eta\,\alpha^{(2)}_{eme} : \mathbf{H_{01}E_{02}}\right], \tag{S8a}$$

$$\mathbf{p_3}^{+,-} = \frac{\varepsilon_0}{\sqrt{I}}\left[\alpha^{(2)}_{eee} : \mathbf{E_{01}E_{02}} - \eta^2\alpha^{(2)}_{emm} : \mathbf{H_{01}H_{02}} - \eta\,\alpha^{(2)}_{eem} : \mathbf{E_{01}H_{02}} + \eta\,\alpha^{(2)}_{eme} : \mathbf{H_{01}E_{02}}\right], \tag{S8b}$$

$$\mathbf{p_3}^{-,+} = \frac{\varepsilon_0}{\sqrt{I}}\left[\alpha^{(2)}_{eee} : \mathbf{E_{01}E_{02}} - \eta^2\alpha^{(2)}_{emm} : \mathbf{H_{01}H_{02}} + \eta\,\alpha^{(2)}_{eem} : \mathbf{E_{01}H_{02}} - \eta\,\alpha^{(2)}_{eme} : \mathbf{H_{01}E_{02}}\right], \tag{S8c}$$

$$\mathbf{p_3}^{-,-} = \frac{\varepsilon_0}{\sqrt{I}}\left[\alpha^{(2)}_{eee} : \mathbf{E_{01}E_{02}} + \eta^2\alpha^{(2)}_{emm} : \mathbf{H_{01}H_{02}} - \eta\,\alpha^{(2)}_{eem} : \mathbf{E_{01}H_{02}} - \eta\,\alpha^{(2)}_{eme} : \mathbf{H_{01}E_{02}}\right]; \tag{S8d}$$

$$\mathbf{m_3}^{+,+} = \frac{1}{\sqrt{I}}\left[\frac{1}{\eta}\alpha^{(2)}_{mee} : \mathbf{E_{01}E_{02}} + \eta\,\alpha^{(2)}_{mmm} : \mathbf{H_{01}H_{02}} + \alpha^{(2)}_{mem} : \mathbf{E_{01}H_{02}} + \alpha^{(2)}_{mme} : \mathbf{H_{01}E_{02}}\right], \tag{S9a}$$

$$\mathbf{m_3}^{+,-} = \frac{1}{\sqrt{I}}\left[\frac{1}{\eta}\alpha^{(2)}_{mee} : \mathbf{E_{01}E_{02}} - \eta\,\alpha^{(2)}_{mmm} : \mathbf{H_{01}H_{02}} - \alpha^{(2)}_{mem} : \mathbf{E_{01}H_{02}} + \alpha^{(2)}_{mme} : \mathbf{H_{01}E_{02}}\right], \tag{S9b}$$

$$\mathbf{m_3}^{-,+} = \frac{1}{\sqrt{I}}\left[\frac{1}{\eta}\alpha^{(2)}_{mee} : \mathbf{E_{01}E_{02}} - \eta\,\alpha^{(2)}_{mmm} : \mathbf{H_{01}H_{02}} + \alpha^{(2)}_{mem} : \mathbf{E_{01}H_{02}} - \alpha^{(2)}_{mme} : \mathbf{H_{01}E_{02}}\right], \tag{S9c}$$

$$\mathbf{m_3}^{-,-} = \frac{1}{\sqrt{I}}\left[\frac{1}{\eta}\alpha^{(2)}_{mee} : \mathbf{E_{01}E_{02}} + \eta\,\alpha^{(2)}_{mmm} : \mathbf{H_{01}H_{02}} - \alpha^{(2)}_{mem} : \mathbf{E_{01}H_{02}} - \alpha^{(2)}_{mme} : \mathbf{H_{01}E_{02}}\right], \tag{S9d}$$

where each of the $\mathbf{p}_3^{+,+}$, $\mathbf{p}_3^{+,-}$ and so on ($\mathbf{m}_3^{+,+}$, $\mathbf{m}_3^{+,-}$, and so on) terms denote the electric (magnetic) dipolar modes induced at frequency ω_3 by the DFG process when using the "+,+", "+,–", etc. pump arrangements. The "1","2", and "3" subscripts denote corresponding vectors at ω_1, ω_2, and ω_3, respectively (i.e., $\mathbf{H_{01}} \equiv \mathbf{H_0}(\omega_1)$, etc.), and the frequency dependence in each of the hyperpolarizabilities has been omitted for compactness. Note that due to the proper nature of the rotation around the x axis used to arrive at various sets in Fig. S4, neither the polar nor the axial effective hyperpolarizability tensors are expected to change sign in any of the set-ups shown in Fig. S4. Hence, the sign before each of the terms in right hand sides in Eqs. (S8) and (S9) follows entirely the sign of the product of the corresponding inducing fundamental fields written in terms of $\mathbf{E}_0$ and $\mathbf{H}_0$. The effective hyperpolarizabilities can be then expressed as following from Eqs. (S8) and (S9) in terms of the electric and magnetic dipolar amplitudes:

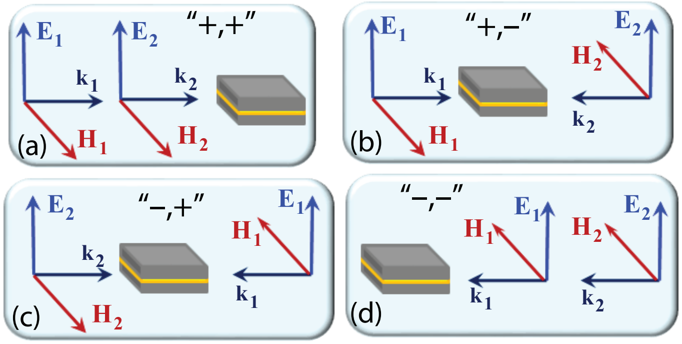

Figure S4. The four pump arrangements used for the effective hyperpolarizabilities retrieval. The "+" ("-") sign denotes the fundamental beam incident in the positive (negative) "z" direction, with the first and second signs denoting the direction of the fundamental waves at ω_1 and ω_2, respectively.

$$\alpha^{(2)}_{eee,xxx} = \frac{0.25}{\varepsilon_0\sqrt{\mathrm{I}}}\left(p^{+,+}_{3x} + p^{+,-}_{3x} + p^{-,+}_{3x} + p^{-,-}_{3x}\right), \tag{S10a}$$

$$\alpha^{(2)}_{emm,xyy} = \frac{0.25}{\varepsilon_0\sqrt{\mathrm{I}}}\left(p^{+,+}_{3x} - p^{+,-}_{3x} - p^{-,+}_{3x} + p^{-,-}_{3x}\right), \tag{S10b}$$

$$\alpha^{(2)}_{eem,xxy} = \frac{0.25}{\varepsilon_0\sqrt{\mathrm{I}}}\left(p^{+,+}_{3x} - p^{+,-}_{3x} + p^{-,+}_{3x} - p^{-,-}_{3x}\right), \tag{S10c}$$

$$\alpha^{(2)}_{eme,xyx} = \frac{0.25}{\varepsilon_0\sqrt{\mathrm{I}}}\left(p^{+,+}_{3x} + p^{+,-}_{3x} - p^{-,+}_{3x} - p^{-,-}_{3x}\right), \tag{S10d}$$

$$\alpha^{(2)}_{mee,yxx} = \frac{\eta}{4\sqrt{\mathrm{I}}}\left(m^{+,+}_{3y} + m^{+,-}_{3y} + m^{-,+}_{3y} + m^{-,-}_{3y}\right), \tag{S11a}$$

$$\alpha^{(2)}_{mmm,yyy} = \frac{\eta}{4\sqrt{\mathrm{I}}}\left(m^{+,+}_{3y} - m^{+,-}_{3y} - m^{-,+}_{3y} + m^{-,-}_{3y}\right), \tag{S11b}$$

$$\alpha^{(2)}_{mem,yxy} = \frac{\eta}{4\sqrt{\mathrm{I}}}\left(m^{+,+}_{3y} - m^{+,-}_{3y} + m^{-,+}_{3y} - m^{-,-}_{3y}\right), \tag{S11c}$$

$$\alpha^{(2)}_{mme,yyx} = \frac{\eta}{4\sqrt{\mathrm{I}}}\left(m^{+,+}_{3y} + m^{+,-}_{3y} - m^{-,+}_{3y} - m^{-,-}_{3y}\right), \tag{S11d}$$

The amplitude of each of the dipolar modes entering the right hand sides of Eqs. (S10) and (S11)) can be retrieved using Eq. (S3), with A, in this case, being the amplitude of the nonlinearly produced field at ω_3 and E_0 being effectively taken as $\sqrt{I}$. Note that this factor cancels with the $\frac{1}{\sqrt{\mathrm{I}}}$ factor in Eqs. (S10) and (S11), so that there is no dependence on I in the retrieved hyperpolarizabilities. Rewritten explicitly in terms of numerical values of the far-field amplitude, the effective hyperpolarizabilities are given by

$$\begin{aligned}
\alpha^{(2)}_{eee,xxx} &= \frac{\pi}{k^2}\left(A^{+,+}_3 + A^{+,-}_3 + A^{-,+}_3 + A^{-,-}_3\right)\Big|_{\substack{\theta=90^\circ\\ \varphi=90^\circ}}, \\
\alpha^{(2)}_{emm,xyy} &= \frac{\pi}{k^2}\left(A^{+,+}_3 - A^{+,-}_3 - A^{-,+}_3 + A^{-,-}_3\right)\Big|_{\substack{\theta=90^\circ\\ \varphi=90^\circ}}, \\
\alpha^{(2)}_{eem,xxy} &= \frac{\pi}{k^2}\left(A^{+,+}_3 - A^{+,-}_3 + A^{-,+}_3 - A^{-,-}_3\right)\Big|_{\substack{\theta=90^\circ\\ \varphi=90^\circ}}, \\
\alpha^{(2)}_{eme,xyx} &= \frac{\pi}{k^2}\left(A^{+,+}_3 + A^{+,-}_3 - A^{-,+}_3 - A^{-,-}_3\right)\Big|_{\substack{\theta=90^\circ\\ \varphi=90^\circ}},
\end{aligned} \tag{S12}$$

$$\begin{aligned}
\alpha^{(2)}_{mee,yxx} &= \frac{\pi}{k^2}\left(A^{+,+}_3 + A^{+,-}_3 + A^{-,+}_3 + A^{-,-}_3\right)\Big|_{\substack{\theta=90^\circ\\ \varphi=0^\circ}}, \\
\alpha^{(2)}_{mmm,yyy} &= \frac{\pi}{k^2}\left(A^{+,+}_3 - A^{+,-}_3 - A^{-,+}_3 + A^{-,-}_3\right)\Big|_{\substack{\theta=90^\circ\\ \varphi=0^\circ}}, \\
\alpha^{(2)}_{mem,yxy} &= \frac{\pi}{k^2}\left(A^{+,+}_3 - A^{+,-}_3 + A^{-,+}_3 - A^{-,-}_3\right)\Big|_{\substack{\theta=90^\circ\\ \varphi=0^\circ}}, \\
\alpha^{(2)}_{mme,yyx} &= \frac{\pi}{k^2}\left(A^{+,+}_3 + A^{+,-}_3 - A^{-,+}_3 - A^{-,-}_3\right)\Big|_{\substack{\theta=90^\circ\\ \varphi=0^\circ}},
\end{aligned} \tag{S13}$$

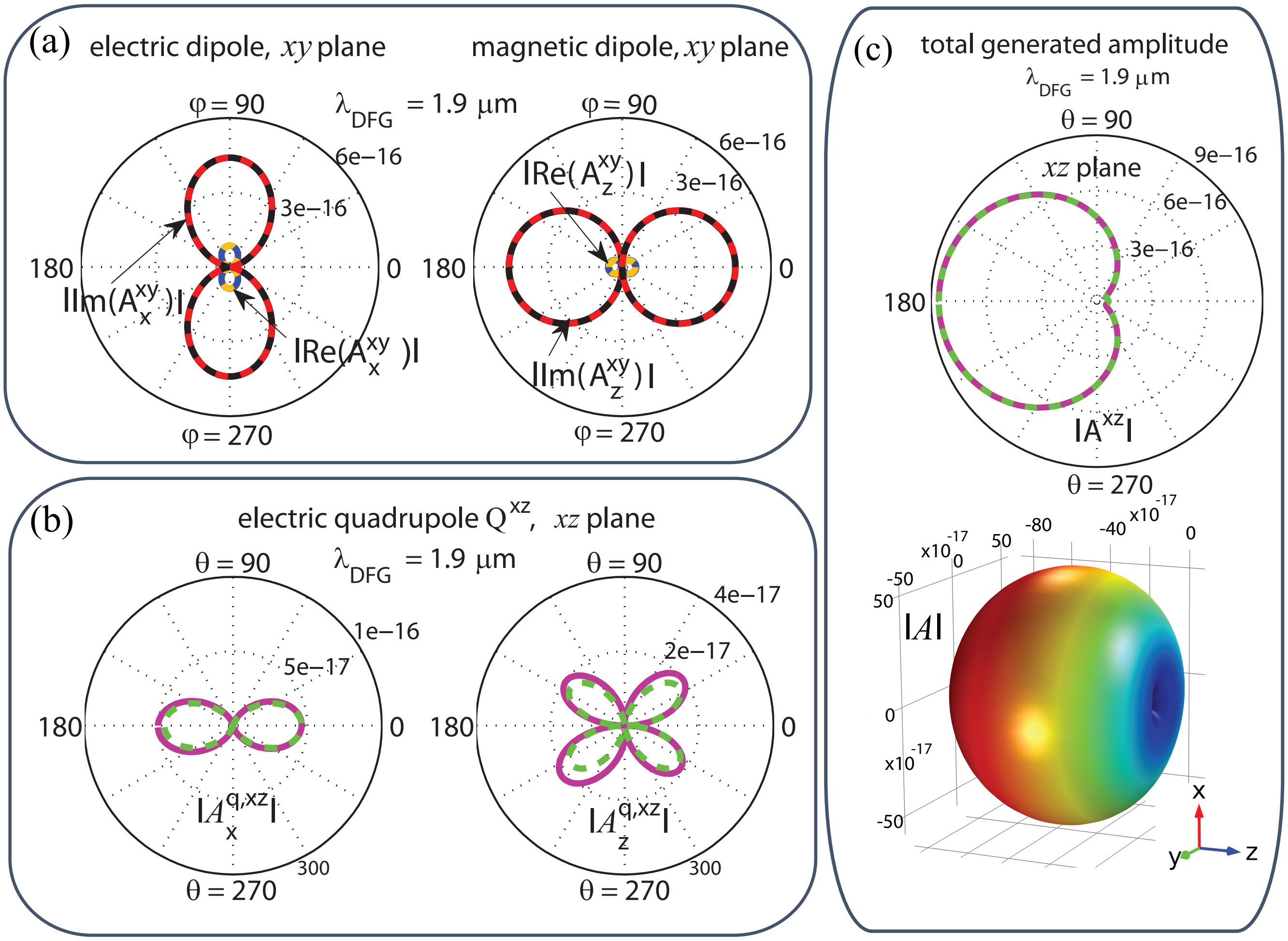

Figure S5. Comparison of numerical (*solid*) and analytical (*dashed*) results similar to that in Figs. 2(b)-2(c), retrieved for the multipolar components in the nonlinearly generated field at 1.9 μm, with the excitation as in Fig. S4(a). The approach similar to that in Figs. S2 and S3 has been used.

where $A_3^{+,+}$, etc. are the far-field amplitudes generated at frequency ω_3 using the corresponding arrangement of fundamental waves from Fig. S4.

The angular dependence comparison shown in Fig. S5(a) thus correspond to the $p^{+,+}$ and $m^{+,+}$ modes. Equation (S12) and (S13) are used in obtaining Fig. 1(c) results.

Note that, alternatively, effective polarizabilities can be retrieved by varying the fundamental beam directions between any other two sets out of the four arrangements shown in Fig. 1(a) that include propagation in the positive and negative z direction: e.g., sets (i) and (iv), (iii) and (ii), or (iii) and (iv). Each combination will produce its own systems of equations equivalent to Eqs. (S4) and (S8), resulting, however, in the same quantitative values for the eight polarizabilities. A similar procedure can be used for nonlinear polarizabilities retrieval for higher order nonlinear responses, only with larger number of equations involved in each of the sets similar to those in Eqs. (S8-S11).

References

1. Poutrina, E. and Urbas, A., J. Opt. **16**, 114005 (2014) (Ref. 49 in the main text).
2. Stratton, J. and L. Chu, L., Phys. Rev. **56**, 99 (1939).
3. Alu, A. and Engheta, N., Phys. Rev. B **79**, 235412 (2009).
4. Evlyukhin, A. B., Reinhardt, C., Zywietz, U., and Chichkov, B. N., Phys. Rev. B **85**, 245411 (1012).
